# Long non-coding RNA and tumor hypoxia: new players ushered toward an old arena

**DOI:** 10.1186/s12929-017-0358-4

**Published:** 2017-08-08

**Authors:** Jing-Wen Shih, Hsing-Jien Kung

**Affiliations:** 1 Ph.D. Program for Cancer Biology and Drug Discovery, College of Medical Science and Technology, Taipei Medical University, Taipei, 110 Taiwan; 2 Research Center of Cancer Translational Medicine, Taipei Medical University, Taipei, 110 Taiwan; 3 Ph.D. Program for Translational Medicine, College of Medical Science and Technology, Taipei Medical University, Taipei, 110 Taiwan; 4 Department of Biochemistry and Molecular Medicine, Comprehensive Cancer Center, University of California at Davis, Sacramento, CA 95817 USA; 5 Institute of Molecular and Genomic Medicine, National Health Research Institutes, Zhunan, Miaoli County 350 Taiwan

**Keywords:** Hypoxia, HIF-1α, Long non-coding RNA, lncRNA, Cancer, Hypoxia-responsive lncRNAs, HRL, Metastasis

## Abstract

Hypoxia is a classic feature of the tumor microenvironment with a profound impact on cancer progression and therapeutic response. Activation of complex hypoxia pathways orchestrated by the transcription factor HIF (hypoxia-inducible factor) contributes to aggressive phenotypes and metastasis in numerous cancers. Over the past few decades, exponentially growing research indicated the importance of the non-coding genome in hypoxic tumor regions. Recently, key roles of long non coding RNAs (lncRNAs) in hypoxia-driven cancer progression have begun to emerge. These hypoxia-responsive lncRNAs (HRLs) play pivotal roles in regulating hypoxic gene expression at chromatic, transcriptional, and post-transcriptional levels by acting as effectors of the indirect response to HIF or direct modulators of the HIF-transcriptional cascade. Notably, the aberrant expression of HRLs significantly correlates with poor outcomes in cancer patients, showing promise for future utility as a tumor marker or therapeutic target. Here we address the latest advances made toward understanding the functional relevance of HRLs, the involvement of these transcripts in hypoxia response and the underlying action mechanisms, highlighting their specific roles in HIF-1 signaling regulation and hypoxia-associated malignant transformation.

**Electronic supplementary material:**

The online version of this article (doi:10.1186/s12929-017-0358-4) contains supplementary material, which is available to authorized users.

## Background

### Tumor hypoxia: An old clinical arena

Maintenance of oxygen homeostasis underlies many critical developmental and physiological processes in mammalian cells. Consequently, to survive under physiological, oxygen deficient (or called hypoxia) conditions, cells have developed an essential and complex set of adaptive mechanisms including the up and down regulation of several hundred protein-coding genes [1]. Hypoxia can occur as a systematic low atmospheric oxygen level or locally at sites of inflammation, tissue ischemia and injury. Of note, hypoxic areas are common mircoenvironmental characteristics of rapidly growing tumors, and the fundamental adaptive responses are believed to be co-opted by cancer cells to promote many key aspects of cancer progression. Indeed, tumor hypoxia is associated with metastases, recurrences and resistance to therapy, and drives critical steps in oncogenic transformation, malignant progression and aggressive phenotype in many cancer types [2–4]. However, the molecular details leading to the hypoxic survival advantage of neoplastic cells have not been fully elucidated. Thus, a better understanding of hypoxic adaption is of critical importance to appreciate tumorigenesis process and to develop novel strategies for pharmacological intervention.

The cellular response to hypoxia insult is mainly governed by a family of transcription factors known as hypoxia inducible factors (HIFs), although HIF-independent responses also exist [5]. HIF is a heterodimer of bHLH-PAS (basic helix-loop-helix DNA binding proteins of the PER-ARNT-SIM family) proteins composed of an oxygen-regulated α subunit and a constitutively expressed, stable β subunit [6]. In mammals, the α subunits are encoded by three genes: *HIF1A*, *HIF2A* (also known as *EPAS1*, Endothelial PAS domain-containing protein 1) and *HIF3A*; whereas the HIF1β subunits (*HIF1B*; also known as *ARNT*, aryl hydrocarbon receptor nuclear translocators), are encoded by two genes *ARNT1* and *ARNT2*. HIF-1α and HIF-2α regulate independent, but overlapping sets of transcriptional target genes, while certain splice variants of HIF-3α exert dominant negative effects on HIF-dependent gene transcription.

**Fig. 1:**
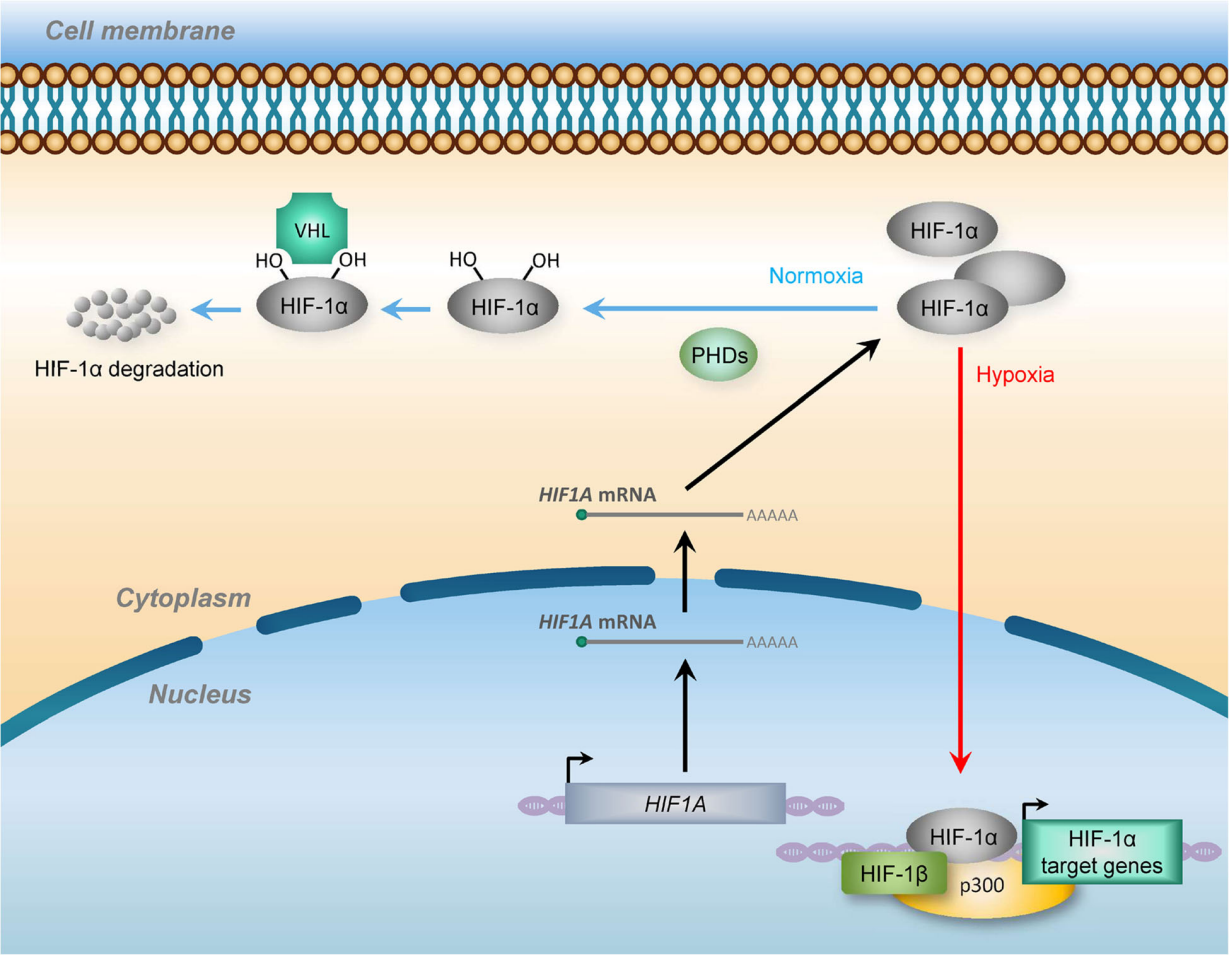
HIF (Hypoxia-inducible factor) activates its downstream target gene transcription in response to hypoxia. Under normoxia, (*blue arrows*), HIF-1α subunits are subjected to hydroxylation by PHDs (prolyl hydroxylase domain proteins) and other prolyl hydroxylases. Hydroxylated HIF-1α subunits are recognized by VHL (von Hippel–Lindau) proteins that target HIF-1α for subsequent ubiquitination and proteasomal degradation. During hypoxia (*red arrows*), the hydroxylation reactions are diminished, resulting in HIF-1α accumulation, dimerization with HIF-1β, binding to target genes and activation of target genes through recruitment of p300 and formation of the transcription initiation complex

Under normoxic conditions, the HIF-1α subunits are rapidly hydroxylated by a family of prolyl hydroxylase domain-containing (PHD1, 2 and 3, also known as Egl-9 family hypoxia inducible factor 1–3, EGLN1–3) dioxygenases, subsequently recognized by the VHL (von Hippel-Lindau tumor suppressor protein) E3 ubiquitin ligase complex, leading to the rapid degradation of HIF-1α through the proteasome pathway (Fig. 1). Upon hypoxia, the dioxygenase PHD activity is inhibited, the HIF-1α subunit is stabilized, accumulating in the nucleus, and forming a stable complex with the β subunit. This complex binds DNA at specific sites containing the consensus nucleotide sequence 5′-(A/G)CGTG-3′ within the hypoxia response elements (HREs) in the promoter regions of HIF1 target genes to stimulate downstream transcription [7]. In particular, it was revealed that, in mammalian systems, HIF-1 can act as an activator or repressor in a cell context-specific manner, whereas the set of genes regulated by HIF-1 differs among cell types [8]. Collectively, hypoxia elicits highly coordinated HIF-dependent transcriptional activation evoking a broad range of cellular adaptions, such as metabolic rewiring, enhanced proliferation, decreased apoptosis, angiogenic growth, and can facilitate cell survival in this scenario [7, 9–11]. The activation of HIF-1 pathways is associated with an aggressive tumor phenotype and poor clinical outcome in numerous cancer types. Therefore, the exploration of the regulatory mechanism underlying HIF-1-mediated transcriptional control may lead to a better understanding of the contribution of hypoxia to tumor progression.

### Long non-coding RNAs: New players in tumorigenesis

In human genome, less than 2% of the DNA sequence is protein-coding, while up to 70% is transcribed into RNA [12, 13]. Many of these transcribed, non-coding RNAs, once thought to represent transcriptional noise, has now been recognized as having important functions in cellular processes. By means of deep sequencing and bioinformatics, an astounding number of noncoding RNAs (ncRNAs) have been annotated in multiple model organisms, from yeast to humans [13–16].

Long non-coding RNAs (lncRNAs) are currently defined as a large and heterogeneous class of regulatory transcripts longer than 200 nucleotides lacking evident protein coding potential [14]. Encompassed within this broad category are different classes of transcribed elements, including long intergenic and intronic ncRNAs, transcribed ultra-conserved regions (TCRs), pseudogenes, enhancer RNAs (eRNAs) and antisense RNAs (asRNAs) [17]. Similar to protein-coding RNAs, the majority of lncRNAs are RNA polymerase II transcripts with a poly-A tail and 5′ cap. Interestingly, most lncRNAs are predominantly localized within the cell nucleus, and exhibit lower evolutionary conservation and lower expression level than mRNAs [14, 16, 18]. According to the GENCODE current release (http://www.gencodegenes.org/stats/current.html) of the Ensembl human genome annotation (GRch38, version 26 from March 2017), 27,720 transcripts originating from 15,787 genes were identified as lncRNAs [19, 20]. Although less than 1% of the identified human lncRNAs have been functionally characterized, these lncRNAs participate in a variety of biological steps, such as chromosome dosage-compensation, imprinting, epigenetic regulation, cell cycle control, nuclear and cytoplasmic trafficking, transcription, translation, splicing and cell differentiation [21–25].

The distinctive biochemical properties of RNA endow these long non-coding RNA transcripts with multi-functionality [26, 27]. For instance, lncRNAs could achieve sequence-specific binding to other nucleic acids simply through one-to-one base pairing interactions, thereby influencing the activities and metabolism of target DNA or RNA. By folding into intricate three dimensional structures providing multifarious interacting surfaces, lncRNA can recognize various molecular targets with high affinity and specificity. In addition, the dynamic RNA expression and structure make lncRNAs well suited for rapid and transient fine-tuning of gene regulation. RNA is relatively flexible and hence provides an excellent platform for evolutionary innovation. More specifically, unrestricted by amino acid-coding potential contained within their sequences, regulatory RNAs are less constrained regarding their conservation. Virtually, RNAs are more tolerant of nucleotide mutations, allowing for the rapid evolution of diverse cellular functions. Most remarkably, as the information recorded within RNA transcripts can be integrated back into the genome through retroviral insertion, these RNA-dependent events could have the capacity to become heritable. Indeed, it has been shown that most lncRNAs exhibit weak primary sequence conservation and elicit functional outcomes through their interactions with cellular macromolecules, such as chromatin DNA, proteins and RNAs [26–30]. The rapidly expanding repertoire of lncRNA functions could be summarized as signals, decoys, guides and scaffolds [28, 30–34]. It is now well appreciated that lncRNA serves as an integral and crucial layer of biological regulation [18, 25, 29, 30].

The spatial- and temporal-specific expression patterns of many identified lncRNAs indicate that the expression of lncRNA is tightly regulated [35, 36]. In recent years, with advancements in global transcriptome profiling, mounting amount of differentially expressed lncRNAs have been linked to various human diseases, most notably cancer [37–40]. To date, numerous cancer-associated lncRNAs are reported to modulate tumor growth, invasion and metastasis. However, only a handful of cancer-associated lncRNAs have been functionally characterized as tumor suppressors or oncogenic drivers in prevalent cancer types [30, 41]. Furthermore, aberrant expression of lncRNAs in cancer marks both clinicopathological features and patient outcomes in a range of cancers. Of note, due to their unique RNA properties, more tissue-specific expression fashion and more stable structure, it is expected that lncRNAs may potentially serve as attractive biomarkers for diagnosis and prognosis. Indeed, several lncRNAs have already been utilized in clinical practice [42]. Thus, knowledge about the functional roles of these aberrantly expressed lncRNAs in tumorigenesis may advance our understanding of cancer progression and unveiled novel diagnostic and therapeutic opportunities.

### New games: Interplay of hypoxia-responsive lncRNAs (HRLs) and HIF signaling in tumorigenesis

Although the regulation of HIF-1 signaling and accompanied global transcriptional responses have been extensively characterized, the downstream and multifarious phenotypic changes associated with hypoxia are still in demand to be further elucidated. Over the last few years, a wealth of independent studies have demonstrated that expression of a specific group of lncRNA molecules are modulated by hypoxia [43–45]. Here, we denote these uniquely regulated long non-coding transcripts as “hypoxia responsive lncRNAs” (HRLs; Table 1 and Additional file 1: Table S1). These RNA molecules are involved in multiple tumorigenesis processes during the hypoxic response, although the underlying mechanisms remain largely unknown.

**Table 1:** List of hypoxia-responsive lncRNAs

lncRNA	Status upon hypoxia	HIF involvement	HRE location	References
*HIF-independent regulation*				
*ATG9B* (noncoding isoform)	Up-regulated	HIF independent	N.D.	[52]
*HIF-mediated direct transcriptional regulation*				
*HOTAIR*	Up-regulated	HIF-1α dependent	ACACG (−1118/−1115)	[53]
*H19*	Up-regulated	HIF-1α dependent	ACGTG (−111/−107)	[54, 71, 72, 122]
			ACGTG (−398/−394)	
			ACGTG (−1434/−1430)	
*UCA1*	Up-regulated	HIF-1α dependent	ACACG (−1515/−1511)	[55, 123]
			ACGTG (−53/−49)	
*NUTF2P3–001*	Up-regulated	HIF-1α dependent	TGGGCGTGGTG (−2114/−2104)	[56]
*EFNA3*	Up-regulated	HIF-1α dependent	ACGTG (−295/−299)	[57]
*HINCUT-1 (uc.475)*	Up-regulated	HIF-1α dependent	GCTCGCATGCGCGG (−527/−514)	[58]
			CACACGTGCGCG (−1233/−1222)	
			CACGTGCGC (−1232/−1224)	
*ANRIL*	Up-regulated	HIF-1α dependent	ACACG (−712/−708)	[59]
*GAPLINC*	Up-regulated	HIF-1α dependent	ACGTG (−320/−324)	[60, 90]
			CACGC (−578/−582)	
*lincRNA-p21*	Up-regulated	HIF-1α dependent & preference	CGTGTACCAC (−797/−788)	[61]
			CGTGAGCCAC (+2603/+2612)	
*aHIF*	Up-regulated	HIF-1α and/or HIF-2α dependent	GCACG (−2045/−2049)	[62, 73, 85]
			GCGTG (−2057/−2061)	
*MALAT1*	Up-regulated	HIF-2α dependent & preference	CGCGCGTGCGCA (−59/−70)	[51, 74, 99, 103, 124]
*NEAT1*	Up-regulated	HIF-2α dependent & preference	Identified by ChIP-Seq	[51, 74, 104, 105]
*lncRNA-SARCC*	VHL-dependent	HIF-2α dependent	ACGTGCTGA (−3258/−3250)	[70]
*HIF-mediated indirect epigenetic regulation*				
*WT1-AS*	Up-regulated	HIF-1 dependent (Indirect: DNA demethylation)	N.D.	[63]
*lncRNA-LET*	Down-regulated	HIF-1α dependent (Indirect: Histone deacetylation)	N.D.	[68, 80]
*Undetermined hypoxia-induced regulation*				
*LncHIFCAR (MIR31HG)*	Up-regulated	HIF-1α dependent	N.D.	[64]
*linc-ROR*	Up-regulated	N.D.	N.D.	[49]
*lncRNA-AK058003*	Up-regulated	N.D.	N.D.	[50]
*NBR2*	Up-regulated	N.D.	N.D.	[65]
*lncRNA-AK123072*	Up-regulated	N.D.	N.D.	[66]
*PVT1*	Up-regulated	N.D.	N.D.	[67]
*ENST00000480739*	Down-regulated	N.D.	N.D.	[69]

Abbreviation: *N.D.* Not determined

Recently, the number of HRLs that have been identified is expanding rapidly, illustrating the complexity of the hypoxia-induced gene reprogramming and the importance to re-consider the involvement of non-coding genome in this adaption. Oxygen deprivation could be acute, transient in local regions, whereas lncRNAs are matchlessly suited to exert a rapid, precise and reversible fine-tuning of the cellular response to this hypoxia stress due to their non-coding and quick biogenesis nature. Indeed, the network of HRLs and their downstream targets are demonstrated to offer the potential for a dynamic biological response to hypoxia. Accordingly, extensive molecular crosstalk between hypoxic signaling cascades and lncRNA metabolism has co-evolved to maintain such exquisite coordination of these adaptive programs.

In the present review, we aim to summarize the most recent findings concerning the tumor hypoxia-associated lncRNAs known to date, with a specific focus on the interplay among these non-coding transcripts and cellular hypoxia response. We describe recent models of molecular mechanisms in which these new players drive hypoxia-induced cancer progression, and attempt to address their relevant potential as clinical biomarker for early diagnosis and prognosis prediction, or even as therapeutic targets in tumor treatment strategies. Our discussion is largely limited to tumor hypoxia, and for roles of lncRNAs in hypoxia- or anoxia-induced cardiovascular and neuromuscular diseases, readers are referred to several excellent recent reviews on these topics [46–48].

## Review

### Regulation of lncRNAs by hypoxia

Given the relevance of HIF pathways on tumor pathogenesis and the pivotal roles of lncRNA in gene expression, it is not surprising that substantial effort has been directed toward defining the transcriptional output of lncRNA in hypoxia-associated malignant progression in the past few years. Increasingly, systematic approaches have been used to identify hypoxia-regulated lncRNAs. By qPCR examination on hypoxic regulation of 89 lncRNAs in hepatocellular cancer (HCC) cells, 20 lncRNAs were identified upregulated >2-fold while 18 downregulated [49]. Microarray analysis of hypoxic gastric cancer cells revealed that 84 lncRNAs were significantly upregulated whereas 70 were downregulated, as compared with normoxic cells [50]. More recently, a systematic analysis coupled RNA-seq with ChIP-seq in hypoxic MCF-7 breast cancer cells demonstrated that lncRNA expression is profoundly regulated by hypoxia. Moreover, the involvement of HIF in the transcriptional response of lncRNAs to hypoxia is far more extensive than previously appreciated [51]. However, in these investigations, the underlying mechanism by which these long noncoding transcripts were regulated upon hypoxia remains largely untouched. Moreover, considering the cell-type and tissue specificity of lncRNA expression, it is expected that there are many more hypoxia-responsive lncRNAs await to be discovered.

Despite these pangenomic studies, a rapidly expanding repository of largely oncogenic lncRNAs have been reported individually to be regulated by hypoxia. Table 1 gives a brief summary to these hypoxia responsive lncRNAs. Among them, *NOS3AS* (the noncoding isoform of *ATG9B*; [52], *HOTAIR* [53], *H19* [54], *UCA1* [55], *NUTF2P3–001* [56], *EFNA3* [57], *HINCUT-1* [58], *ANRIL* [59], *GAPLINC* [60], *linc-p21* [61], *aHIF* [62], *MALAT1* [51], *NEAT1* [51], *WT1-AS* [63], *LncHIFCAR* [64], *linc-ROR* [49], *lncRNA-AK058003* [50], *NBR2* [65], *lncRNA-AK123072* [66] and *PVT1* [67] are up-regulated, whereas *lncRNA-LET* [68] and *ENST00000480739* [69] are down-regulated in response to hypoxia. Interestingly, *lncRNA-SARCC* could differentially respond to hypoxia in a VHL-dependent manner [70], while the regulatory mechanisms underlying the hypoxic altered expression of several HRLs still remained uncharacterized (Table 1).

It is well appreciated that the HIF complex is a crucial transcription factor coordinating cellular transcriptional response under hypoxic stress. According to their interplay with the HIF complex, the hypoxia responsive lncRNAs can be categorized into HIF-dependent and HIF-independent.

#### HIF-independent regulation of hypoxia responsive lncRNA

An antisense transcript of *eNOS* (endothelial nitric-oxide synthase) mRNA, the noncoding isoform of *ATG9B* (also known as eNOS antisense [*NOS3AS*] or autophagy 9-like 2 [*APG9L2*]) induced by hypoxia, is the only one to date reported to be HIF-independent [52]. The hypoxic induction of this antisense transcript is mainly through transcript stabilization which in turn leads to eNOS expression suppression [52]. However, its involvement in tumorigenesis and even whether it acts as an RNA or a protein have not been completely elucidated [52].

#### HIF-mediated direct regulation of hypoxia responsive lncRNA transcription

In addition, a number of HIF-dependent HRLs have been studied in detail. Similarly with miRNAs and protein-coding genes, HIF could directly regulate lncRNAs at transcriptional level through HREs (hypoxia response elements) that usually reside within their promoter regions. By in silico prediction combined with biochemical assays such as ChIP (chromatin immunoprecipitation) and EMSA (electrophoretic mobility shift assay), a set of HREs responsible for hypoxic induction of lncRNAs have been identified (Table 1).

For instance, the promoters of *HOTAIR*, *H19*, *lncRNA-UCA1*, *lncRNA-NUTF2P3–001*, *lncRNA-EFNA3, HINCUTs*, *ANRIL, NEAT1, MALAT1* and *GAPLINC* are demonstrated to harbor functional HREs required for HIF-1α-mediated transcriptional activation under hypoxia. Notably, the interplay between HIF-1 and other transcription factors can impact on the induction of HRLs upon hypoxia. For instance, *H19* expression is not affected in wild type *p53* cells, whereas it is dramatically upregulated in *p53* null cells, revealing that *p53* status may govern lncRNA expression under hypoxia [71] Meanwhile, in glioblastoma, the process of HIF-1α binding to the H19 promoter requires *SP1* (specific protein 1) to be activated by HIF-1α [72]. Also, *PTEN* status is a critical factor affecting hypoxia-induced H19 level in multiple GBM cell lines and human clinical specimens [72]. In addition to HIF-1α, systematic ChIP-seq data of human hypoxic breast cancer cells revealed that, HIF-2α also bound with lncRNA promoters and even in a higher proportion than HIF-1α, suggesting a pivotal role of HIF-2α in the regulation of the long noncoding transcriptome upon hypoxia [50]. Indeed, both HIF-1α and HIF-2α proteins were shown to bind the HRE in the human *aHIF* gene specifically [73]. Notably, like hypoxia-responsive coding genes and miRNAs, both HIF-1α and HIF-2α regulate distinct but overlapping sets of lncRNAs. As an example, silencing of HIF-1α, but not HIF-2α, significantly attenuated hypoxia-induced *lincRNA-p21* upregulation [61]. Nonetheless, in the cases of *NEAT1* and *MALAT1* (also known as NEAT2) lncRNAs, the loss of function assays showed that HIF-2α, rather than HIF-1α, is the preferred transcriptional activator in MCF-7 cells [74]. Most intriguingly, lncRNA-SARCC (Suppressing Androgen Receptor in Renal Cell Carcinoma) could differentially respond to hypoxia in a von Hippel-Lindau (VHL)-dependent manner [70]. It was shown that LncRNA-SARCC can be transcriptionally suppressed by HIF-2α through HREs within its promoter. Moreover, the same study revealed a LncRNA-SARCC-mediated post-transcriptional regulation of androgen receptor (AR) by physically binding and de-stabilizing AR protein to suppress AR/HIF-2alpha/C-MYC signals. Along these lines, the negative feedback modulation between LncRNA-SARCC/AR complex and HIF-2α signaling may thereby contribute to differential RCC progression in a VHL-dependent manner [70]. Collectively, this work demonstrates that the hypoxia-responsive lncRNAs can be regulated at transcription level directly by HIF-1α and/or HIF-2α in a variety of cancers.

#### HIF-mediated indirect regulation of hypoxia responsive lncRNA transcription

Currently, several studies have described hypoxic induction of lncRNAs through mechanisms without the direct involvement of HIF on their promoters. These indirect regulation seems to be achieved through epigenetic mechanisms. Not surprisingly, as an integral hypoxic transcription factor, the HIF complex transactivates the expression of multiple genes, including those involved in epigenetic modifications among HDACs (histone deacetylases) tested in hepatocellular carcinoma SMMC-7721 cells in a recent study, only HDAC3 was specifically upregulated by hypoxic insult [68]. This hypoxia-induced HDAC3 suppressed lncRNA-LET expression by reducing the levels of histone H3 and H4 acetylation in the lncRNA-LET promoter region [68], revealing an indirect regulation mediated by HIF-1α.

In myeloid leukemia cells, another antisense RNA *WT1-AS* was reported to be regulated upon hypoxia in a similar way [63]. *WT1-AS* lncRNA is transcribed antisense from Intron 1 or from the same promoter region of *WT1*. Hypoxia provokes a decrease in expression and activity of DNMT1 (DNA methyltransferase 1) as well as an increase in expression and activity of the evolutionarily conserved dioxygenases TET2 (Ten-Eleven-Translocation 2) and TET3 (Ten-Eleven-Translocation 3), resulting in demethylation of the CpG island in Intron 1 of *WT1* gene. This loss of methylation in *WT1* Intron 1 CpG island therefore led to upregulation of both *WT1-AS* lncRNA and *WT1* mRNA [63].

It is noteworthy that, in both cases of *lncRNA-LET* and *WT1-AS*, the hypoxia induced-modulation of lncRNA expression could be reverted by HIF-1α knockdown, indicating the critical involvement of HIF-1 in these hypoxia-caused lncRNA expression alteration [63, 68]. Also, these findings together demonstrated that epigenetic modification of chromatin plays an important role in HIF-mediated indirect modulation on lncRNA expression under hypoxia.

### Regulation of hypoxia signaling by lncRNAs

To date, most of the HRLs are functionally characterized with profound impact on tumorigenesis in a spectrum of cancer types. However, the vast majority of HRLs described have not yet been studied in mechanistic detail, while the few that have been mechanistically investigated might provide clues regarding their biological roles in response to hypoxia. An overview of the tumor hypoxia-associated lncRNAs is shown in Table 2, categorized according to their underlying action mechanisms. Some HRLs directly affect HIF activity, while others may have indirect regulations. Notably, several HRLs might adapt more than one action mechanism. The accumulating discovery of novel HRLs with more thorough investigation will surely reveal additional scenarios.

**Table 2:** Hypoxia-associated lncRNA-mediated HIF signaling control and cancer progression

lncRNA	Cancer Types	Clinical association	Regulatory effect	Mechanism	Refs
*LncRNAs modulating HIF pathway*					
*ENST00000480739*	Pancreatic ductal adenocarcinoma	• Downregulation in PDAC • Expression negatively associated with tumor node metastasis (TNM) stage and lymph node metastasis • Independent risk factor for PDAC survival following surgery.	↓ Invasion ↑ *OS-9* mRNA & protein	**Transcriptional regulation** (*ENST00000480739* induces *OS-9* expression at the transcriptional level)	[69, 77]
*RERT-lncRNA*	Hepatocellular carcinoma	The expression levels of *RERT-lncRNA* and EGLN2 were significantly correlated in HCC	↑ EGLN2 expression	**Transcriptional regulation** (*RERT-lncRNA* induces *EGLN2/PHD1* expression at the transcriptional level)	[78]
*HIF2PUT*	Osteosarcoma	Expression of *HIF2PUT* is correlated with *HIF2A* mRNA	↓ Cell proliferation and migration ↓ Expression of CSC marker CD133 ↓ Sphere-forming ability	**Transcriptional regulation** (*HIF2A* mRNA expression was co-regulated with *HIF2PUT expression*)	[79]
*LncHIFCAR*	Oral cancer	• Upregulation in oral cancer • High levels of *LncHIFCAR* predicted worse overall survival and recurrence-free survival	↑ Hypoxic glycolysis ↑ Tumor growth	**Transcriptional regulation** (*LncHIFCAR* acts as HIF-1α coactivator)	[64]
*lncRNA-LET*	Lung squamous-cell cancer, hepatocellular Carcinoma and colorectal cancer	Downregulation in LSCC, HCC and CRC	↓ Metastasis ↓ Invasion	**mRNA stability control** (The association between *lncRNA-LET* and NF90 protein enhanced the degradation of NF90, thereby decreasing *HIF1A* mRNA)	[68, 80]
*HIF1A-AS2*	Renal cell carcinoma, Glioblastoma	Upregulation in non- papillary clear-cell renal carcinoma and glioblastoma	↑ Growth of M-GSCs ↑ Neurosphere-forming capacity of M-GSCs ↑ Glioblastoma tumor growth	**mRNA stability control** (Binding of *HIF1A-AS2* to the *HIF1A* mRNA 3′-UTR could expose AU-rich elements and thus increase the degradation of *HIF1A* mRNA)	[62, 73, 82, 85]
				**Scaffold of RNP complex** (The direct interaction among the *HIF1A-AS2* interactome, IGF2BP2 and DHX9 is needed for maintenance of expression of their target gene, *HMGA1*)	
*linc-ROR*	Hepatocellular cancer	N.D.	↑ Cell viability during hypoxia ↑ Tumor growth	**Sequestration of miRNAs** (Downregulation of miR145-mediated repression of P70S6K1 expression)	[49]
*PVT1*	Gastric cancer	• Upregulation in TNBC • High expression levels of PVT1 correlated with advanced tumor stage and lymph node metastasis	↑ GC cell proliferation ↑ GC cell invasion	**Sequestration of miRNAs** (Downregulation of miR-186-mediated repression of HIF-1α expression)	[89]
*lincRNA-p21*	Cervical, lung and breast cancer cell lines	N.D.	↑ Hypoxic glycolysis ↑ Tumor growth	**Protein-Protein Interaction Decoy** (Stabilization of HIF-1α by disrupting the VHL-HIF-1α Interaction)	[61]
*LINK-A*	Triple-negative breast cancer	• Upregulation in TNBC • High levels of *LINK-A* correlated with unfavorable recurrence-free survival for breast cancer patients	↑ Glycolysis ↑ Tumor growth	**Scaffold of RNP complex** (*LINK-A* facilitates the recruitment of BRK and BRK kinase activation, thereby causing HIF-1α stabilization, HIF-1α/p300 interaction, and activation of HIF-1α transcriptional programs under normoxic conditions)	[96]
*LncRNAs modulating hypoxia response through a peripheral mechanism rather than directly on HIF pathway*					
*WT1-AS*	Myeloid Leukemia	• Upregulation in Wilms’ tumors • Aberrant WT1-AS splicing often found in acute myeloid leukemia	N.D.	**Epigenetic regulation** (*WT1-AS* mediate hypoxia-induced *WT-1* mRNA upregulation through modulating histone methylation)	[63, 125]
*lncRNA-AK058003*	Gastric cancer	Upregulation in GC	↑ Invasion & migration ↑ Metastasis	**Epigenetic regulation** (*AK058003* expression is positively correlated with *SNCG* expression and *SNCG* promoter demethylation)	[50]
*HINCUT-1 (uc.475)*	Colon and breast cancer cell lines	N.D.	↑ Hypoxic cell proliferation	**Transcriptional regulation** (*HINCUT-1* is required for the expression of *OGT* mRNA expression and global O-GlcNAcylation of proteins)	[58]
*GAPLINC*	Gastric cancer	• Upregulation in GC • High expression of *GAPLINC* correlates with poorer survival • GAPLINC correlates with CD44 activation in GC tissues	↑ Proliferation ↓ Apoptosis ↑ Invasion ↑ Migration	**Sequestration of miRNAs** (Downregulation of miR-211–3p-mediated repression of CD44)	[60, 90]
*lncRNA-EFNA3*	Breast cancer	A strong correlation between high *EFNA3* expression and shorter metastasis-free survival in breast cancer patients	↑ Cell extravasation ↑ Metastatic dissemination	**Sequestration of miRNAs** (Downregulation of miR-210-mediated repression of EFNA3)	[57]
*NUTF2P3–001*	Pancreatic cancer	• Upregulation in pancreatic cancer • A positive correlation between lncRNA-NUTF2P3–001 and KRAS, which is associated with advanced tumor stage and worse prognosis.	↑ Cell viability, proliferation ↑ Invasion ↑ KRAS expression ↑ Metastasis	**Sequestration of miRNAs** (Downregulation of miR-3923-mediated repression of KRAS)	[56]
*H19*	Glioblastoma, hepatocellular carcinoma, bladder cancer and a large group of tumors	• Increased expression of *H19* RNA is shown in a large group of tumors • Upregulation in HCC cancer • *H19* overexpression confers a poor prognosis for GBM patients	↑ Hypoxia-driven invasion & migration ↑ Tumor growth	**Sequestration of miRNAs** (Downregulation of miR-181d-mediated repression of β-catenin expression)	[54, 71, 72, 122]
				**Transcriptional regulation** (Activation of downstream target gene involved in angiogenesis, survival and tumorigenesis through unclear mechanism)	
*lncRNA-SARCC*	Renal cell carcinoma	Differentially regulated by hypoxia in a von Hippel-Lindau (VHL)-dependent manner in RCC clinical specimens.	↑ Hypoxic cell cycle progression (VHL-restored RCC cells) ↓ Hypoxic cell cycle progression (VHL-mutant RCC cells)	**Control of protein activity** (*LncRNA-SARCC* can post-transcriptionally regulate AR by physically binding and destabilizing AR protein to suppress AR/HIF-2α/C-MYC signals)	[70]
*MALAT1*	Breast cancer, neuroblastoma, hepatocellular carcinoma, HUVECs	Associated with poor clinical outcome in multiple cancers	↑ Cell growth ↑ Glycolysis ↑ Migration & invasion ↑ Vasculature formation ↑ Metastasis	**Scaffold of RNP complex** (Affects recruitment of splicing factors to the nuclear speckles and splicing patterns of alternative exons)	[51, 74, 99, 102, 103, 122, 124, 126]
				**Protein-Protein Interaction Decoy** (Causes disassociation of the VHL protein from HIF-1α/HIF-2α)	
*NEAT1*	Breast cancer	High expression of *NEAT1* is associated with poor survival of breast cancer patients	↑ Proliferation ↓ Apoptosis ↑ Clonogenic survival ↑ Paraspeckle formation	**Scaffold of RNP complex** (Induces paraspeckle formation, thereby enhancing cancer cell survival in hypoxia)	[51, 74, 104, 105]
*LncRNAs modulating hypoxia response* via *unclear mechanisms*					
*HOTAIR*	Non-small cell lung carcinoma	High level of *HOTAIR* is associated with poor clinical outcome in multiple cancers	↑ Cell proliferation under hypoxia ↑ Invasion & migration under hypoxia ↓ Apoptosis under hypoxia	**Unclear mechanism** (Possibly through epigenetic modification)	[53, 127]
*ANRIL*	Osteosarcoma	Upregulation in osteosarcoma	↑ Hypoxic viability ↑ Hypoxia-induced Invasion ↓ Hypoxia-induced apoptosis	**Unclear mechanism** (Possibly through epigenetic modification)	[59]
*UCA1*	Bladder cancer	• Upregulation in bladder cancer • *UCA1* expression associated with the clinical stage and histologic grade of bladder cancer	↑ Cell proliferation under hypoxia ↑ Invasion & migration under hypoxia ↓ Apoptosis under hypoxia	**Unclear mechanism**	[55, 123]
*PVT1*	Cervical Cancer	• Upregulation in ICC tissue • High expression of PVT1 correlates with poorer overall survival	↑ Cell proliferation ↑ Migration and invasion ↓ Apoptosis ↑ Cisplatin resistance	**Unclear mechanism** (Possible involvement of the interaction with Nucleolin)	[67]

Abbreviation: *CRC* colorectal cancer, *CSC* cancer stem cell, *GC* Gastric cancer, *HCC* hepatocellular cancer, *HUVECs* Human umbilical vein endothelial cells, *ICC* Immunocytochemistry, *LC* lung cancer, *M-GSCs* Mesenchymal glioblastoma multiforme stem-like cells, *N.D.* Not determined, *NSCLC* nonsmall cell lung carcinoma, *OSCC* Oral squamous cell carcinoma, *PDAC* pancreatic ductal adenocarcinoma, *RCC* Renal Cell Carcinoma, *RNP* ribonucleic protein, *VHL* von Hippel-Lindau protein

#### HRLs in epigenetic regulation

One of the major recurring theme in lncRNA biology is their ability to recruit protein factors for regulation of chromatin states. This class of lncRNAs often function as critical *cis*- and *trans*-acting modulators of protein-coding gene expression. Recent genome-wide studies of RNA–protein interactions have demonstrated that chromatin-modifying complexes interact with a series of lncRNAs, suggesting that these transcripts might direct the recruitment of protein complex to specific genomic loci. For example, lncRNAs such as *HOTAIR* (HOX transcript antisense RNA) and *ANRIL* (also known as CDKN2B antisense RNA 1) have even been shown to interact with more than one histone-modifying complex, and therefore been proposed to coordinate the targeting of histone-modifying complexes [75]. Of note, these two antisense RNAs, *HOTAIR* and *ANRIL,* have been shown to be hypoxia-inducible and direct transcriptional targets of HIF, contributing to aggressive phenotype in lung cancer and osteosarcoma cells, respectively [53, 59]. However, whether these two asRNAs modulate hypoxic gene expression through epigenetic modification remains to be determined.

In addition, in myeloid leukemia cells, another hypoxia-inducible antisense RNA, *WT1-AS* lncRNA, was reported to modulate histone methylation *in cis* at the *WT1* mRNA transcription start site [63]. *WT1* gene encodes a transcription factor WT1, which is overexpressed in a wide variety of tumor types, including acute myeloid leukemia (AML). Interestingly, *WT1-AS* lncRNA is transcribed antisense to *WT1* and observed to interact with sense *WT1* mRNA, resulting in WT1 protein up-regulation [76]. Moreover, *WT1-AS* lncRNA could mediate hypoxia-induced *WT-1* mRNA upregulation through modulating histone methylation H3K4me3 and H3K9me3 at the *WT1* mRNA transcription start site, thus contributing to tumor progression [63]. This study showed that the hypoxia-sensitive histone methylation changes in the *WT1* locus are dependent upon *WT1-AS* lncRNA expression, and provided another model of HRP-mediated epigenetic regulatory mechanism.

Notably, in gastric cancer cells, *lncRNA-AK058003*, located upstream of synuclein gamma (*SNCG*, a metastasis-associated protein), is robustly upregulated by hypoxia and regulates *SNCG* expression *in cis* by demethylating CpG islands within its promoter, thereby promoting hypoxia-induced metastasis [50].

#### HRLs in transcriptional regulation

**Fig. 2:**
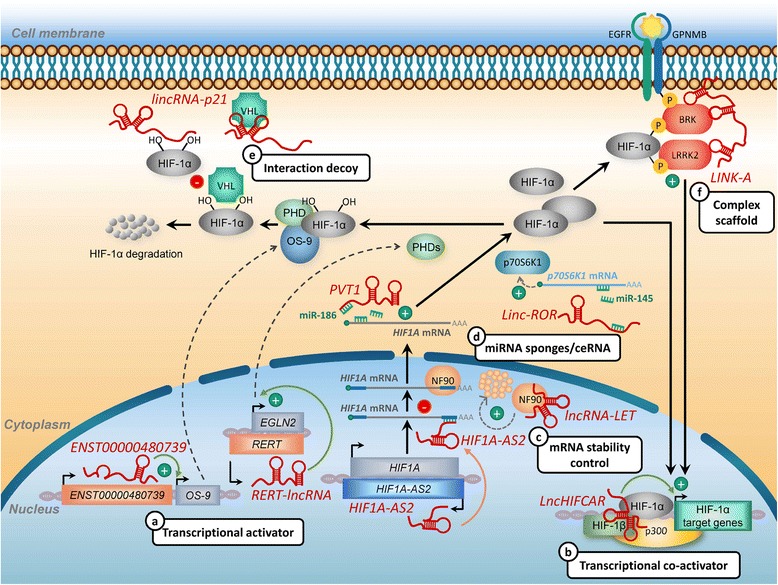
Mechanisms of hypoxia-reponsive lncRNAs affecting HIF-1α activity. **a** Transcriptional activator. lncRNA *ENST00000480739* suppresses HIF-1α expression by upregulating *OS-9* transcription. OS-9 is a HIF-1α-binding protein that facilitates HIF-1α hydroxylation and proteasomal degradation. Another lncRNA *RERT-lncRNA* decreases the HIF-1α levels by upregulation of *EGLN2* mRNA. *EGLN2* encodes prolyl hydroxylase PHD1, which is responsible for HIF-1α hydroxylation and promoting HIF-1α degradation. **b** Transcriptional co-activator. *LncHIFCAR* acts as an oncogenic HIF-1α co-activator through direct binding to HIF-1α, thereby facilitating the recruitment of HIF-1α and p300 cofactor to the target promoters and stimulating HIF-1 target gene expression. **c** mRNA stability control. *lncRNA-LET* interacts with RNA-binding protein NF90, which has been implicated in the stabilization of target mRNAs. As the association between *lncRNA-LET* and NF90 could enhance the degradation of NF90, the hypoxia-induced downregulation of *lncRNA-LET* may thereby increase *HIF-1A* mRNA stability under hypoxic conditions. In addition, lncRNA *HIF1A-AS2*, an antisense transcripts transcribed from the 3′-UTR of the sense *HIF1A* mRNA negatively regulates *HIF1A* mRNA expression. Through base-pair binding to the *HIF-1A* mRNA 3′-UTR, *HIF1A-AS2* could expose AU-rich elements within the *HIF-1A* mRNA 3′-UTR, thereby destabilizing of *HIF-1A* mRNA. **d** miRNA sponge/ceRNA. miR-145 negatively regulates expression of p70S6K1, a protein kinase responsible for promoting protein synthesis. Hypoxia-induced *lincRNA-ROR* could upregulate HIF-1α expression by sequestering endogenous miR-145. In addition to *lincRNA-ROR*, *PVT1* lncRNAs upregulate HIF-1α expression by sponging miR-186. **e** Interaction decoy. *lincRNA-p21* is capable of binding to both VHL and HIF-1α, leading to disruption of VHL/HIF-1α interaction. **f** *LINK-A* recruits and activates BRK and LRRK2 to phosphorylates HIF-1α. These phosphorylation modifications prevents HIF-1α degradation under normoxia and facilitates the interaction between HIF-1α and cofactor p300, thereby activating HIF-1 target genes. See text for detailed discussion

Aside from remodeling chromatin structure, several HRLs are found to modulate transcription and thereby affecting HIF activity via multiple mechanisms. For example, HRLs can influence the transcription of HIF regulatory factors and fine-tune the HIF network in an indirect way. In human PDAC (pancreatic ductal adenocarcinoma) cells, a novel lncRNA *ENST00000480739* was identified to target HIF-1α expression by upregulating OS-9 (osteosarcoma amplified-9) mRNA expression [69]. OS-9 is a HIF-1α-binding protein mediating destabilization of HIF-1α by promoting the interaction between HIF-1α and PHD (proline hydroxylase domain) proteins, which in turn facilitates HIF-1α hydroxylation and proteasomal degradation [77] (Fig. 2a). Interestingly, *ENST00000480739* is located upstream of the *OS-9* promoter region and acts *in cis* to induce its transcription, leading to HIF-1α downregulation *in trans* and suppression of tumor cell invasion [69]. Of note, *ENST00000480739* expression level was remarkably decreased in PDAC, negatively associated with lymph node metastasis and an independent prognostic factor of PDAC patient survival following surgery [69], consistent with its regulatory role in HIF-1α-driven tumor metastasis and progression (Fig. 2a, Table 2).

Likewise, *RERT-lncRNA* overlaps with *EGLN2* that encodes prolyl hydroxylase PHD1 responsible for HIF-1α hydroxylation and degradation (Fig. 2a). In HCC (hepatocelluar carcinoma) cells, *RERT-lncRNA* was reported to suppress the HIF-1α expression by upregulation of *EGLN2* at the transcriptional level [78]. Although the expression of *RERT-lncRNA* in low-oxygen conditions has not been addressed in this study, it provides an example of lncRNA indirectly modulating HIF pathway through regulating EGLN2 expression (Fig. 2a, Table 2).

*HIF2PUT* (HIF-2α promoter upstream transcript, previously known as TCONS_00004241) is another example of hypoxia-associated lncRNA acting *in* *cis* [79]. *HIF2PUT* was identified as an antisense lncRNA transcribed upstream of the promotor region of *HIF2A* mRNA. The lncRNAs of this category are known as PROMPTs (promoter upstream transcripts), which appear to modulate the transcriptional expression of host protein-coding genes. Indeed, in primary osteosarcoma tumors and cancer cell lines, *HIF2PUT* expression was co-regulated with *HIF2A* mRNA, whereas *HIF2PUT* expression alteration led to a parallel variation in *HIF2A* mRNA expression [79]. However, the expression and role of *HIF2PUT* under hypoxia remained to be elucidated.

Interestingly, an ultra-conserved transcript, *HINCUT-1* (hypoxia-induced noncoding ultra-conserved transcript 1, also named as uc.475), is transcribed as a retained intron of *OGT* (O-linked N-acetylglucosamine transferase) mRNA [58]. Being a direct transcriptional target of HIF-1α, *HINCUT-1* is overexpressed in colon and breast cancer cells under hypoxia and in primary colorectal tumors [58]. As *HINCUT-1* knockdown reduced *OGT* gene and protein expression, it is suggested that *HINCUT-1* transcript has a transcriptional activity [58]. Intriguingly, the *HINCUT-1* DNA region was demonstrated to exhibit an enhancer-like activity both under hypoxia and normoxia, revealing its multifaceted regulatory roles in gene transcription [58].

Moreover, HRLs could act directly on specific transcription factors. More recently, we identified *LncHIFCAR* (long noncoding HIF-1α co-activating RNA, previously known as *MIR31HG*) as another hypoxia-inducible lncRNA. *LncHIFCAR* acts as a critical mediator in hypoxia-associated tumorigenesis steps, including hypoxic cell growth, metabolic regulation, stem-like properties and metastatic potential in oral cancer [64]. Mechanistically, we described a distinct mechanism, independent of the stabilization or translocation of HIF-1α, in which *LncHIFCAR* serves as an oncogenic HIF-1α co-activator through direct binding to HIF-1α, thereby facilitating the recruitment of HIF-1α and p300 cofactor to the target promoters. The increased abundance of the HIF complex work in concert to further stimulate the hypoxia-induced HIF-1 transcriptional reprogramming and subsequent cancer progression [64]. Remarkably, our results also uncovered the up-regulation of *LncHIFCAR* in oral carcinoma and the clinical relevance of *LncHIFCAR* as an independent adverse prognostic predictor for the cancer progression [64] (Fig. 2b, Table 2). Notably, with the extraordinary variety of transcriptional regulatory mechanisms discussed here, it is expected that additional means of lncRNAs adapted for fine-tuning hypoxia-induced transcriptional regulation are likely to be uncovered in the near future.

#### HRLs in post-transcriptional control

Once transcribed, eukaryotic mRNAs are subjected to a series of post-transcriptional control of gene expression, including mRNA decay and miRNA-mediated repression. Accumulating data shows that, during hypoxia, these post-transcriptional events could be centrally controlled by HRLs.

##### mRNA stability control

Two HRLs *lncRNA-LET* (Long noncoding RNA Low Expression in Tumor) and *HIF1A-AS2* (*HIF1A Antisense RNA 2*; also known as *aHIF*) have both been implicated in both negative regulation of *HIF1A* mRNA stability. Unlike most of hypoxia-responsive lncRNAs reported, *lncRNA-LET* expression is suppressed upon hypoxia due to activation of HDAC3 [68] as mentioned above. *lncRNA-LET* was found to be generally downregulated in various types of tumors, including hepatocellular carcinomas, colorectal cancers, squamous-cell lung carcinomas and gallbladder cancer [68, 80]. Mechanistically, *lncRNA-LET* could interact with RNA-binding protein NF90 (nuclear factor 90), which has been implicated in the stabilization of several target mRNAs [81]. As the association between *lncRNA-LET* and NF90 was shown to enhance the degradation of NF90 [68], the hypoxia-induced downregulation of *lncRNA-LET* may thereby increase *HIF-1A* mRNA stability under hypoxic conditions and lead to hypoxia-induced cancer cell invasion (Fig. 2c, Table 2). Notably, decreased expression of *lncRNA-LET* was significantly correlated with tumor micrometastases and encapsulation in HCC [68], while the similar association with low differentiated histology, advanced tumor status, and nodal status was also reported in primary gallbladder cancer [80]. Multivariate analysis revealed *lncRNA-LET* as an independent prognostic marker for metastasis and death in gallbladder cancer patients [80].

In addition, lncRNA *HIF1A-AS2*, an antisense transcripts transcribed from the 3′-UTR (untranslated region) of the sense *HIF1A* mRNA, was found to be hypoxia-inducible [62], upregulated in various tumors [62, 82–85] and negatively regulates *HIF1A* mRNA expression [73, 82]. More specifically, through base-pair binding to the *HIF-1A* mRNA 3′-UTR, *HIF1A-AS2* could expose AU-rich elements within the *HIF-1A* mRNA 3′-UTR, thereby destabilizing of *HIF-1A* mRNA [82] (Fig. 2c, Table 2). More recently, lncRNA *HIF1A-AS2* was also found to be selectively induced in mesenchymal GSCs (glioblastoma stem-like cells) in response to hypoxia [85]. *HIF1A-AS2* promotes tumorigenicity and required for hypoxia-dependent molecular reprogramming in GSCs [85]. Other than impact on RNA stability, *HIF1A-AS2* could enhance the expression of several of mRNA targets, such as *HMGA1* (high mobility group AT-hook 1) mRNA, and subsequent molecular response to hypoxic stress through the direct interaction with IGF2BP2 (insulin-like growth factor 2 mRNA-binding protein 2) and DHX9 (ATP-dependent RNA helicase A) [85], which suggests lncRNA *HIF1A-AS2* may modulate gene expression via multiple mechanisms.

##### miRNA sponges

Functional manipulations of HRLs have demonstrated that several HRLs have roles in hypoxia response by acting as a ‘competitive endogenous RNA (ceRNAs)’ network and by participating in endogenous miRNA ‘sponges’ or ‘decoys’ to interfere with miRNA-mediated mRNA destabilization. In recent years, it has been postulated that a genome-wide regulatory network exists between coding and non-coding RNAs that manifests through competition for miRNA binding. A number of mammalian lncRNAs, including *lincRNA-ROR, PVT1, GAPLINC, EFNA3, H19* and *NUTF2P3–001* have miRNA-binding sites and therefore act as “miRNA sponges” to sequester miRNAs away from their mRNA targets (Table 2). Specifically, in HCC cells, hypoxia-induced *lincRNA-ROR* could upregulate HIF-1α expression by sequestering endogenous miR-145 [49]. Remarkably, miR-145 inhibits *p70S6K1* post-transcriptional expression by targeting its 3′-UTR, whereas the downstream molecules of p70S6K1, HIF-1, is also simultaneously decreased upon miR-145 overexpression. [86]. Accordingly, by nullifying miR-145, *lincRNA-ROR* indirectly activates HIF-1α expression (Fig. 2d, Table 2) [49].

In addition to *lincRNA-ROR*, *PVT1* lncRNA also functions most likely in this way. In cervical cancer, *PVT1* expression is shown to be upregulated in response to hypoxia and immune response stimulation [67]. This transcript has been designated as an oncogenic role due to its contribution to the aggressive phenotype of multiple cancers, including cervical cancer and gastric cancer [67, 87–89]. In gastric cancer cells, PVT1 was demonstrated to upregulate HIF-1α expression by sponging miR-186 to revert miR-186-mediated repression of HIF-1α expression and consequentially promote GC cell progression indirectly [89] (Fig. 2d, Table 2).

Likewise, some of the HRLs may regulate other hypoxia-associated proteins than HIF-1α via functioning as a ceRNA. A typical example among this kind of HRLs is *GAPLINC* (Gastric Adenocarcinoma Associated, Positive CD44 Regulator, Long Intergenic Non-Coding RNA). In gastric cancer, *GAPLINC* is directly activated by HIF-1α and its overexpression is associated with poor prognosis and could promote tumor migration and invasive behavior [60]. Echoing these results, *GAPLINC* was shown to enhance tumor migration and invasion by acting as a molecular decoy for miR-211–3p to rescue CD44 expression [90].

Aside from *GAPLINC*, *EFNA3* lncRNA was shown to be induced in hypoxic breast cancer cells and also to function in this way [57]. *EFNA3* lncRNAs are transcribed from the *EFNA3* locus which also encoded Ephrin-A3, a signaling ligand crucial for tumor metastasis and extravasation. *EFNA3* lncRNAs refer to 4 different isoform according to different transcription start sites and truncated 3′-UTR [57]. Upon hypoxia, hypoxia-induced miR-210 can target 3′-UTR of *EFNA3* mRNA and prevent its translation of [91–93]. More interestingly, with the same 3′-UTR region, *EFNA3* lncRNAs could increase *EFNA3* mRNA translation by depleting miR-210 [57], revealing a mechanism by which hypoxia-induced *EFNA3* lncRNAs act as ceRNAs that relieve EFNA3 mRNA from miRNA repression allowing for efficient transcription during hypoxia.

In pancreatic cancer, *lncRNA-NUTF2P3–001* is identified as a hypoxia-inducible gene and a HIF-1α direct transcriptional downstream target [56]. Overexpression of *lncRNA-NUTF2P3–001* is found in pancreatic cancer tissue and associated with advanced tumor stage and poor prognosis [56]. Similarly, *lncRNA-NUTF2P3–001* has been suggested to act as a ‘sponge’ that inhibits miR-3923 that targeted KRAS, a critical driver in pancreatic cancer tumorigenesis [56].

In particular, lncRNA H19, the mammalian imprinted transcript, was among the first reported hypoxia-regulated lncRNAs [54]. Upon hypoxia, HIF-1α-dependent H19 expression is robustly upregulated in hepatocellular and bladder carcinoma [54]. In the past few years, H19 has been implicated as an oncogene in various cancers including gastric, bladder, colorectal and breast cancers, and its action mechanism can be dissected into two major categories: a reservoir of miR-675 that suppresses its targets, and a modulator of miRNAs or protein factors through their interactions [94]. More recently, in glioblastoma, hypoxia-induced H19 upregulation was reported to drive an aggressive phenotype by absorbing miR-181d and aborting the miR-181d-mediated repression on β-catenin, an oncogenic factor associated with EMT (epithelial-mesenchymal transition) [72].

Collectively, it seems quite feasible that the long and small non-coding RNA systems would eventually intertwine to form mutually reciprocal repression feedback networks. In this scenario, any alteration in the expression level of one member in this network, and accordingly the amount of miRNAs associated with it, would disturb the overall accessible pool of ncRNAs, resulting in concordant shift to transcriptional reprogramming. To delineate this new layer of post-transcriptional regulation directed by hypoxia responsive ceRNAs, it will be of interest to further explore the relevant co-working relationships between lncRNAs and miRNAs under hypoxia.

#### HRLs in control of protein activity and/or higher-order complex formation

Apart from HRLs-exerted modulation of gene expression events through interplay with other RNAs, HRLs can also act on the protein level. It is well known that RNA transcripts could associate with proteins to form ribonucleoprotein particles (RNPs) to exert their functions. To date, our knowledge of the lncRNA-containing RNPs is relatively limited compared to those composed of other RNAs, such as rRNAs and snRNAs. Along these lines, lncRNA/protein interaction network in cells could provide delicate means to fine-tune gene expression. For instance, as mentioned above, *LncRNA-SARCC* could exert post-transcriptional regulation of AR through physical interaction and de-stabilizing AR protein to repress AR/HIF-2α/C-MYC signaling [70]. In cervical cancer, hypoxia-inducible lncRNA *PVT1* (plasmacytoma variant translocation 1) was suggested to exert its oncogenic role through association with the multifunctional protein, Nucleolin [67]. Nevertheless, there are several reports showing that, in addition to modulating single protein activity, HRLs can also act as decoys or scaffolds to elicit direct impacts on the formation of higher-order RNP complexes.

*LincRNA-p21*, directly activated by HIF-1α at transcriptional level in low-oxygen conditions, provides an example to illustrate the complex formation [61]. *LincRNA-p21* was capable of binding to both VHL and HIF-1α, resulting in disrupting the VHL/HIF-1α interaction. Given that VHL-binding is essential for hydroxylation of the HIF-1α protein and the following degradation via the ubiquitin-proteasome pathway, *lincRNA-p21-*driven disassociation of VHL from HIF-1α by binding to each of them could thereby attenuate VHL-mediated HIF-1α ubiquitination and increase HIF-1α accumulation under hypoxia (Fig. 2e, Table 2) [61]. Being a direct transcriptional target of HIF-1α, *lincRNA-p21* itself generates a positive feedback loop promoting HIF-dependent pathways such as hypoxic glycolysis and Warburg effect [61]. Also noted, *lincRNA-p21* was first identified as a key repressor in p53-dependent transcriptional responses through the physical association with hnRNP-K (heterogeneous nuclear ribonucleoprotein K), which is necessary for accurate genomic localization of hnRNPK at repressed gene locus and control of p53-driven apoptosis [95]. Together, these results suggest, through association with different protein factors, one lncRNA may initiate diverse transcriptional programs.

In TNBC (triple-negative breast cancer), a highly prognostic lncRNA, *LINK-A* (long intergenic non-coding RNA for kinase activation) plays a pivotal role for the growth factor-induced normoxic HIF1 signaling [96], although its expression upon hypoxia remains to be determined. *LINK-A* interacts with two protein kinases BRK (breast tumor kinase) and LRRK2 (leucine-rich repeat kinase 2). This RNP complex could transfer signal from EGFR (epidermal growth factor receptor):GPNMB (transmembrane glycoprotein NMB) membrane heterodimer receptor to the nucleus by phosphorylation of HIF-1α (Fig. 2f, Table 2). More specifically, upon receiving signal triggered by HB-EGF (heparin-binding EGF), *LINK-A* recruits BRK and LRRK2 to EGFR:GPNMB heterodimer, thereby inducing conformation changes and activating their kinase activities. The activated BRK and LRRK2 phosphorylates HIF-1α at Tyr 565 and Ser 797, respectively. Notably, Tyr 565 phosphorylation inhibits adjacent Pro 564 hydroxylation, which prevents HIF-1α degradation under normoxia, while Ser 797 phosphorylation facilitates the interaction between HIF-1α and cofactor p300, activating HIF-1 target genes in response to HB-EGF stimulation [96]. Both *LINK-A* expression and the activation of HIF-1 signaling correlate well with TNBC, promoting glycolysis reprogramming, angiogenesis and tumorigenesis in breast cancer [96].

In many studies, *MALAT1* (metastasis-associated lung adenocarcinoma transcript 1, also known as *NEAT2*) and *NEAT1* (nuclear enriched abundant transcript 1) are another two hypoxia-induced lncRNAs via HIF binding to their promoter regions (Table 1). Both of them are highly conserved lncRNAs that localize to specific nuclear compartments [97] and are expressed from adjacent single exon genes on chromosome 11q [51]. *MALAT1* is an intensively-studied lncRNA and frequently upregulated in various cancers, such as breast cancer, lung cancer and colon cancer, in which it promotes cellular proliferation, angiogenesis, tumor growth and metastasis [98–100], implying its potential as a cancer biomarker and a promising target for cancer therapy.

*MALAT1* is a nuclear-retained lncRNA, implicating its function in nuclear processes, such as nuclear architecture organization, splicing, and gene expression. Indeed, *MALAT1* is a core component of the nuclear speckles (also known as SC35 splicing domains or interchromatin granule clusters). As these nuclear structures are enriched with poly-adenylated RNAs and factors involved in mRNA processing, splicing, and export, the localization of *MALAT1* reveals a role in RNA metabolism [101]. Though the overall assembly of nuclear speckles is not dependent on the attendance of *MALAT1*, *MALAT1* associates with splicing factors and is required for their recruitment to the nuclear speckles, where they affect the alternative splicing patterns of exons [97, 100]. In addition, other studies have shown that *MALAT1* regulates alternative splicing by modulating phosphorylation of pre-mRNA splicing factors [102]. However, its role in regulating RNA splicing during hypoxia remains to be determined. Recently, *MALAT1* has also been linked to the transcriptional and/or posttranscriptional control of gene expression via interactions with a series of specific factors, such as transcriptional coactivators, histone methyltransferases/demethylases and pre-mRNA splicing factors [100]. Also, one recent study suggested that, in hepatic cells, arsenite-induced *MALAT1* could enhance the disassociation of VHL from HIF-1α, attenuating VHL-mediated HIF-1α ubiquitination, which in turn causes HIF-1α accumulation and arsenite-induced glycolysis [103]. Collectively, to interpret the varying findings in different model systems, it is proposed that the function of *MALAT1* is determined by the combination of interacting factors [100]. Feasibly, associations with divergent protein partners or nucleic acids could augment the spectrum of *MALAT1* functions. Otherwise, *MALAT1* may function as a “nuclear reservoir” for capture and storage of RNAs and their associated proteins.

In contrast to its neighboring lncRNA *MALAT1* which does not significantly affect the formation of the nuclear speckles, lncRNA *NEAT1* is an essential architectural component for nuclear paraspeckle assembly [97, 100]. The exact biological function of paraspeckles is currently unclear, but implicated in gene expression through the retention of hyper-edited RNA transcripts and other multifunctional protein components in paraspeckles to prevent their potential translation and activity [104]. Particularly, the hypoxic induction of nuclear paraspeckles leads to the nuclear retention of one such transcript, *F11R* (F11 receptor, also known as junctional adhesion molecule 1, JAM1) mRNA which was shown to be subjected to A-to-I editing [105, 106]. However, suppression of *NEAT1* abrogated hypoxia-induced nuclear retention of *F11R* mRNA, conferring a role of *NEAT1* in nuclear retention of mRNAs during hypoxia [105]. Notably, induction of NEAT1 in hypoxia also leads to hallmarks of increased tumorigenesis, including acceleration of cell proliferation, promotion of cell survival, and reduced apoptosis [105]. Moreover, in patients with breast cancer and prostate cancer, high tumor *NEAT1* expression correlates with poor survival [105, 107]. As both *MALAT1* and *NEAT1* are implicated in control of nuclear structures, it will be interesting to further explore the extent to which these hypoxia-induced nuclear structures contribute to the regulation of other hypoxia-responsive genes.

## Conclusion and future perspectives

The cellular adaption in response to hypoxia requires the precisely orchestrated regulatory network. The aforementioned discussion has provided a summary of our current knowledge regarding the expression, functions and mechanisms of hypoxia-responsive lncRNAs (HRLs). These novel mechanisms reveal a predominant, unanticipated role of HRLs in the control of gene expression under hypoxia and illustrates the elaborate network among the different classes of RNAs, including lncRNAs, miRNAs and mRNAs. Indeed, it has been reported that HIF is a major regulator of the coding and non-coding transcriptome [51, 108]. On the contrary, some lncRNAs described above regulate HIF activity and stabilization, adding an additional layer of complexity to the role of lncRNAs in gene expression control during hypoxia. Therefore, identification and characterization of the complete set of non-coding transcripts involved in the adaptation to limited oxygen supply is of utmost relevance for a deeper understanding of hypoxia-associated tumorigenesis steps.

The emergence of HRLs as important elements of cellular adaptation to hypoxia has prompted intense interest in elucidating how hypoxia controls HRL expression. The vast majority of studies have focused on the hypoxia-induced altered transcript abundance of HRLs. It is worth noting that many HRLs express multiple splice isoforms. As our understanding of HRLs advances, it will be equally important to uncover the hypoxia-mediated modulatory effects on HRLs at other levels of biogenesis. Recent work has demonstrated the association between hypoxia and alternative spicing mechanisms that may have a direct impact on HRL expression [109]. It is also revealed that hypoxia could induce adenosine-to-inosine (A-to-I) RNA sequence editing [110]. In addition, a wealth of evidence indicate that RNA bioactivity and stability are influenced by interactions with RNA binding proteins and/or RNA helicases [111, 112]. These additional levels of post-transcriptional control may also participate in the distinctive shifts in HRL activity patterns upon low oxygen tension.

One particularly well-characterized mechanism, the so-called “ceRNA (competing endogenous RNA)” or “RNA sponges”, by which HRLs regulate gene expression involves the interaction with miRNAs in a manner that can sequester these molecules and reduce their inhibitory effect on target mRNA (Fig. 2d, Table 2). Of note, it is postulated that ceRNA mechanism can only work under certain appropriate stoichiometry between miRNAs and ceRNA [113]. Therefore, beyond the presence of matching miRNA seed sequences, experimental evidence and knockout studies are required to further corroborate ceRNA mechanisms. Additionally, thorough investigation is needed to decipher the complex regulatory relationships existing among hypoxia-regulated lncRNAs and miRNAs. Target affinity of HRLs for a single miRNA or even a group of miRNAs may be modulated upon hypoxia. Given that numerous lncRNAs are host of miRNAs, the presence of poly-cistronic non-coding genes along with genomic miRNA clusters and overlapping targets suggest that conjunctional control is a fundamental theme of non-coding RNA biology. Currently, better in silico tools are necessary to predict integrated ceRNA effects during cellular response to hypoxia. Together, addressing these unsolved concerns regarding HRL biology will directly advance our knowledge of their role in adaptive responses and hypoxia-induced tumorigenesis.

Hypoxia-inducible factor (HIF) is central in coordinating HRL expression during exposure to low oxygen levels. Notably, mounting evidence implicates a number of other transcription factors and HIF-independent pathways involving p53, NF-κB, mTOR, endoplasmic reticulum (ER) stress and the unfolded protein response (UPR) play critical complementary roles in hypoxic transcriptional shift of coding genes [114, 115]. Consistent with this notion, lncRNA H19 expression under hypoxia is dependent on the status of *p53* [71], *SP1* and *PTEN* [72], whereas the expression of *lncRNA-SARCC* could differentially respond to hypoxia in a VHL-dependent manner [70]. The possible involvement of other transcription factors and pathways may account for the widespread context dependency of hypoxia-mediated regulation on HRL expression, such as the different expression patterns of lncRNA *HIF2CUT* [79, 116] and *HIF1A-AS2* [62] in a variety of cancers. This context dependence would surely have vital implications for the consideration of HRLs for possible applications. Therefore, in-depth future investigation is required to characterize the molecular details of how hypoxia elicts divergent effects on the expression of select subsets of HRLs.

Given the prominent biological and pathological roles played by HRLs in hypoxia-driven cancer progression, these transcripts may be exploited as valuable indicators of the intrinsic characteristics of the tumors, or as novel prognostic tools to aid in disease outcomes prediction, cancer patient assessment and management (Table 2). Moreover, considering the prevalence of hypoxia-induced metastasis, these findings suggest that individual HRLs or RNA panels combined with the expression signatures of prevailing mRNA, HRL and miRNA could potentially be utilized as tailored biomarkers for monitoring tumor hypoxia and metastasis. Compared with the current DNA, mRNA, or protein biomarkers, using lncRNA as biomarker for cancer diagnosis and prognosis has been shown to hold several advantages in light of its sensitivity, specificity, stability and easy accessibility [41]. Most remarkably, lncRNAs can be easily detected from the body fluid such as saliva, urine or blood [117]. Accordingly, the genome-wide identification and functional annotation of tissue-specific HRL signatures in conjunction with their expression patterns in tumors hold promising potential for the development of accurate, non-invasive biomarkers for early tumor detection and prognosis prediction. Nevertheless, in the past decade, GWASs (Genome-wide association study) have become a mainstream way to identify germline SNPs that may predispose individuals to cancer. Notably, structural approaches have revealed that even SNPs can change local RNA structure at functionally relevant sites required for miRNA or protein binding [118]. Although the relationship between HRLs and SNPs remains largely elusive currently, the identification of HRLs harboring cancer-associated SNPs by the use of GWAS data may help to categorize patient populations at risk of cancer, disease phenotypes and patient outcomes. Hence, the clinical translation of HRL-related SNPs in GWAS data may warrant future research attention relates to cancer epidemiology.

For decades, HIF-1 and its downstream effectors that mediate tumor metabolic adaptation, have been long recognized as potential targets for cancer drug due to their profound impacts in cancer progression. However, considering the complexity of the HIF-1α signaling network and the requirements of targeting protein-protein interactions, it becomes extremely challenging to design HIF-1α inhibitors. Recently, HRLs has been increasingly considered as potential cancer therapeutic targets owing to their tissue specificity, high expression levels and crucial roles in tumor growth and progression [41]. Till now, the development of RNA-targeting methods has provided tremendous opportunities to modulate lncRNAs for cancer therapy [119, 120]. Most excitingly, novel classes of RNA-based therapeutics show great potential to modulate lncRNA activity in diverse ways [121]. Splice-switching oligonucleotides could redirect alternative splicing to restore gene function. Moreover, steric blocking oligonucleotides or small molecule could impede the interaction between lncRNAs and their binding partners. Although most lncRNA-targeted treatments remains in the early stages of development, future technical innovations along with better insights into lncRNA pathways in cancer biology will provide new opportunities. With in-depth characterization of both hypoxic microenvironment and non-coding RNA network, more hypoxia-responsive lncRNAs will be undoubtedly exploited as targets and stimulate development of ideal therapeutics for tumor patients in the near future.

## Additional file

Additional file 1:
**Table S1.** Hypoxia-responsive lncRNAs. (DOCX 19 kb)

## Abbreviations

ANRIL: Antisense RNA in the INK4 locus
BRK: Breast tumor kinase
ceRNA: Competitive endogenous RNA
CRC: Colorectal cancer
CSC: Cancer stem cell
DHX9: ATP-dependent RNA helicase A
EGFR: Epidermal growth factor receptor
EMT: Epithelial-mesenchymal transition
ER: Endoplasmic reticulum
GAPLINC: Gastric adenocarcinoma associated, positive CD44 regulator, long intergenic non-coding RNA
GC: Gastric cancer
GPNMB: Transmembrane glycoprotein NMB
GSC: Glioblastoma stem-like cells
GWAS: Genome-wide association study
H19: Imprinted maternally expressed transcript
HB-EGF: Heparin-binding EGF
HCC: Hepatocellular cancer
HIF1A-AS2: HIF1A Antisense RNA 2
HIF2PUT: HIF-2α promoter upstream transcript
HINCUT-1: Hypoxia-induced noncoding ultra-conserved transcript 1
HMGA1: High mobility group AT-hook 1
HOTAIR: HOX transcript antisense RNA
HUVECs: Human umbilical vein endothelial cells
ICC: Immunocytochemistry
IGF2BP2: Insulin-like growth factor 2 mRNA-binding protein 2
LC: Lung cancer
linc-ROR: Long intergenic non-protein coding RNA, regulator of reprogramming
LINK-A: Long intergenic non-coding RNA for kinase activation
LncHIFCAR: Long noncoding HIF-1α co-activating RNA
lncRNA: Long non-coding RNA
lncRNA-LET: Long noncoding RNA Low Expression in Tumor
lncRNA-SARCC: LncRNA-Suppressing Androgen Receptorin Renal Cell Carcinoma
LRRK2: Leucine-rich repeat kinase 2
MALAT1: Metastasis associated lung adenocarcinoma transcript 1
M-GSCs: Mesenchymal glioblastoma multiforme stem-like cells
N.D.: Not determined
NBR2: Neighbor Of BRCA1 Gene 2
ncRNA: Non-coding RNA
NEAT1: Nuclear paraspeckle assembly transcript 1
NF90: Nuclear factor 90
NSCLC: Non-small cell lung carcinoma
OGT: O-linked N-acetylglucosamine transferase
OS-9: Osteosarcoma amplified-9
OSCC: Oral squamous cell carcinoma
PDAC: Pancreatic ductal adenocarcinoma
PROMPTs: Promoter upstream transcripts
PVT1: Plasmacytoma variant translocation 1
RCC: Renal Cell Carcinoma
RNP: Ribonucleic protein
SNCG: Synuclein gamma
TNBC: Triple-negative breast cancer
UCA1: Urothelial cancer associated 1
UPR: Unfolded protein response
UTR: Untranslated region
VHL: von Hippel-Lindau protein
