# Supplementary material for: Long non-coding RNA and tumor hypoxia: new players ushered toward an old arena
Source: J Biomed Sci. 2017 Aug 8;24:53. doi: 10.1186/s12929-017-0358-4 (PMC5547530; doi:10.1186/s12929-017-0358-4)
Supplement: Additional file 1: — Table S1. Hypoxia-responsive lncRNAs. (DOCX 19 kb) [file 12929_2017_358_MOESM1_ESM.docx]

**Table S1 | Hypoxia-responsive lncRNAs**

| lncRNA | Synonyms | Accession number  (Ensembl version) | |
| --- | --- | --- | --- |
| *ATG9B* (noncoding isoform) | *Autophagy Related 9B, Autophagy Related 9 Homolog B, APG9L2, SONE, Nitric Oxide Synthase 3 Antisense(NOS3AS), FLJ14885* | NR_073169.1/NR_133652.1  ENSG00000181652 | |
| *GAPLINC* | *Gastric adenocarcinoma predictive long intergenic noncoding RNA, LINC01540, long intergenic non-protein coding RNA 1540, TCONS_00026238* | NR_110429  ENSG00000266835 | |
| *lncRNA-AK058003* |  | GenBank: AK058003.1 | |
| *aHIF* | *HIF1A-AS2 (HIF1A antisense RNA 2), 3'aHIF-1A* | NR_045406  (ENSG00000258667) | |
| *linc-ROR* | *lincRNA-RoR, lincRNA-ST8SIA3, ROR* | NR_048536  (ENSG00000258609) | |
| *lincRNA-p21* | *TP53COR1 (tumor protein p53 pathway corepressor 1), linc-p21, lincRNA-p21, Trp53cor1, Ak144811, Gm16197* | GenBank: CD515754.1 | |
| *LncHIFCAR* | *long noncoding HIF-1α co-activating RNA, hsa-lnc-31, LOC554202, MIR31HG, MIR31 host gene* | NR_027054  (ENSG00000171889) | |
| *lncRNA-EFNA3* | *EFNA3-002*/*EFNA3-003* | EFNA3-002(ENST00000498667.1)  EFNA3-003(ENST00000470294.5) | |
| *MALAT1* | *PRO1073, NEAT2, mascRNA, NCRNA00047, HCN, PRO2853, MALAT-1, LINC00047* | NR_002819  (ENSG00000251562) | |
| *NEAT1* | *LINC00084, "long intergenic non-protein coding RNA 84", MENepsilon/beta, "nuclear enriched abundant transcript 1", TncRNA, "trophoblast derived non-protein coding RNA", VINC, "virus inducible non-coding RNA"* | NR_028272  (ENSG00000245532) | |
| *NBR2* | *Neighbor Of BRCA1 Gene 2, Non-Protein Coding RNA 192, NCRNA00192* | NR_003108.2, NR_138145.1 (ENSG00000198496) | |
| *HINCUT-1* | *hypoxia-induced noncoding ultraconserved transcript 1, uc.475* | Intron 4 of NM_181672  (ENSG00000147162) | |
| *HOTAIR* | *HOXAS, HOXC-AS4, HOXC11-AS1, NCRNA00072* | NR_003716  (ENSG00000228630) | |
| *H19* | *ASM, ASM1, D11S813E, LINC00008, long intergenic non-protein coding RNA 8, NCRNA00008, non-protein coding RNA 8* | NR_002196  (ENSG00000130600) | |
| *UCA1* | *Urothelial cancer associated 1, CUDR, LINC00178, NCRNA00178, UCAT1, onco-lncRNA-36* | | NR_015379  (ENSG00000214049) |
| *NUTF2P3-001* | *NUTF2P3, nuclear transport factor 2 pseudogene 3, LINC00079, onco-lncRNA-100, NCRNA00079* | NG_044239  (ENSG00000228248) | |
| *WT1-AS* | *WT1 Antisense RNA, Wilms Tumor Upstream Neighbor 1, WIT-1, WIT1, Wilms Tumor-Associated Antisense RNA, WT1-AS1, WT1AS* | ENSG00000183242 | |
| *ANRIL* | *CDKN2B-AS1 (CDKN2B antisense RNA 1), CDKN2B-AS, CDKN2BAS, NCRNA00089, "non-protein coding RNA 89", "p15 antisense RNA", p15AS, PCAT12, "prostate cancer associated transcript 12", RP11-145E5.4* | NR_003529  ENSG00000240498 | |
| *PVT1* | *LINC00079, "long intergenic non-protein coding RNA 79", NCRNA00079, "non-protein coding RNA 79", onco-lncRNA-100, Plasmacytoma Variant Translocation 1, Long Intergenic Non-Protein Coding RNA 79* | NR_003367  ENSG00000249859 | |
| *ENST00000480739* | *RPL13AP23 (ribosomal protein L13a pseudogene 23), RP 11–571M6.1* | ENST00000480739  NG_010224  (ENSG00000242990) | |
| *lncRNA-LET* | *NPTN-IT1 (NPTN intronic transcript 1)* | NR_103844  (ENSG00000281183) | |
| *lncRNA-SARCC* | *Suppressing Androgen Receptor in Renal Cell Carcinoma* | ENST00000460407 | |
| *RERT-lncRNA* | *RAB4B-EGLN2, RAB4B-EGLN2 read-through long non-coding RNA, RAB4B-EGLN2 Readthrough* | NR_037791  (ENSG00000171570) | |
| *LINK-A* | *LOC339535, LINC01139 (long intergenic non-protein coding RNA 1139)* | NR_015407  ENSG00000215808 | |
| *HIF2PUT* | *TCONS_00004241* | TCONS_00004241 (USCS website) | |
